# DNA Methylation Pattern as Important Epigenetic Criterion in Cancer

**DOI:** 10.1155/2013/317569

**Published:** 2013-12-23

**Authors:** Mehrdad Ghavifekr Fakhr, Majid Farshdousti Hagh, Dariush Shanehbandi, Behzad Baradaran

**Affiliations:** Immunology Research Center, Tabriz University of Medical Sciences, Tabriz, Iran

## Abstract

Epigenetic modifications can affect the long-term gene expression without any change in nucleotide sequence of the DNA. Epigenetic processes intervene in the cell differentiation, chromatin structure, and activity of genes since the embryonic period. However, disorders in genes' epigenetic pattern can affect the mechanisms such as cell division, apoptosis, and response to the environmental stimuli which may lead to the incidence of different diseases and cancers. Since epigenetic changes may return to their natural state, they could be used as important targets in the treatment of cancer and similar malignancies. The aim of this review is to assess the epigenetic changes in normal and cancerous cells, the causative factors, and epigenetic therapies and treatments.

## 1. Introduction

All somatic cells possess the same genotype, since they have originated from the growth and division of a common progenitor cell. However, during the differentiation process cells become specialized and obtain a variety of functions and features by expressing and suppressing different sets of genes. Normally these settings are controlled by epigenetic processes. The genetics of changes and cell division is heritable. Epigenetic features are changed during the tumor induction and cancer development with different patterns and characteristics [1].

## 2. Background

The term *epigenetics* is made of two parts: Greek prefix “*epi*” which means upon or over and “*Genetics*” which is the science of genes, heredity, and variations in living organisms. It defines what is occurring in the physical state of the genes and chromatin. This word was first defined by Conrad Hal Waddington as the interaction between genes and their environment that creates the phenotype emphasizing that epigenetic mechanisms are different in response to a given environment. Waddington later pointed out that one of the main characteristics of epigenetic changes will occur in gene expression without any mutations. The nongenetic manifestation of traits in morphology had been introduced by Lamarck many years before Waddington propounded this idea. In this new definition, epigenetics is referred to as those changes in the genes functions which are transmitted through both mitosis and meiosis without causing any alterations in the DNA sequence [2].

## 3. Epigenetic Mechanisms

Epigenetic regulations are derived from the fact that the DNA packaging in the nucleus affects the genes expression directly [3]. In general, the increased condensation of DNA enhances the probability of genes silencing. In return, decreasing compression of DNA leads to its accessibility for transcription machinery and increased expression of genes. Physically, the genome in the eukaryotic cells is packed in chromatin structure which determines its accessibility for functions such as transcription, replication, and DNA repair [4]. In general, three common biochemical mechanisms occur in the cell for epigenetic changes: DNA methylation, histone changes, and association of nonhistone proteins such as Polycomb and Trithorax complexes.

## 4. DNA Methylation

**Figure 1:**
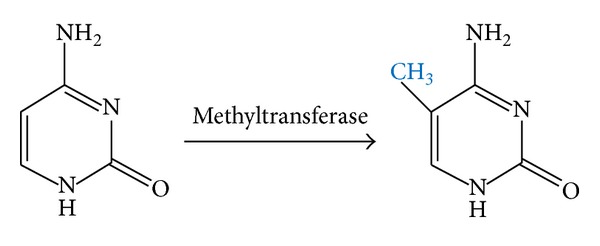
Methylation of cytosine in carbon 5.

In mammals, DNA methylation is a common epigenetic change in DNA. After DNA synthesis, cytosines within the dinucleotide CpGs are methylated at their carbon 5 by DNA methyltransferase (DNMT) (Figure 1). CpGs which undergo methylation could be found either in singular situation or in clusters so-called CpG islands [5]. But if the methylation happens in the promoter region of the genes, it would likely lead to gene silencing [6]. Normally, long-term silencing of genes occurs only in X-linked, imprinted, and germ-cell specific genes. CpG islands of DNA sequence that contain plenty of C and G nucleotides are commonly hypermethylated in tumor cells which could result in silencing of tumor suppressor genes [7].

An important step toward understanding the role of DNA methylation is to specify its location in the genome. Nowadays, this can be achieved by utilizing methods developed for genome-wide mapping of 5 mc (5 methylcytosine) such as microarrays or high-throughput sequencing [8].

Data obtained from methylation studies show that cytosine methylation is available throughout the genome of mammals. Moreover, in most of the genomes in which DNA has lower CpG content, there is a high degree of cytosine methylation while, CpG islands often remain nonmethylated [9, 10].

## 5. Histone Changes 

**Figure 2:**
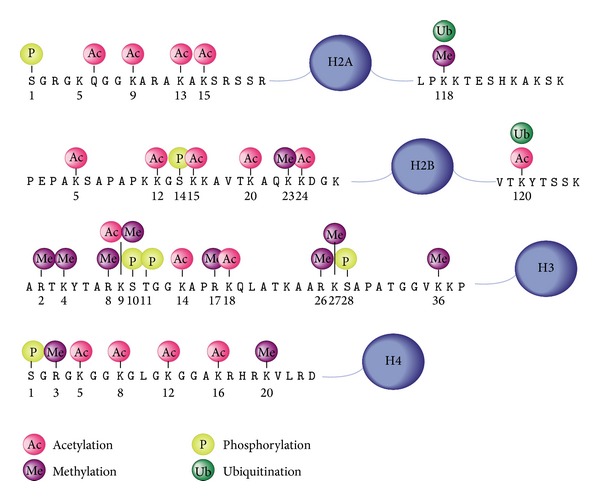
Epigenetic modifications of histone amino acids [77].

Histone changes include posttranslation modifications in the histone proteins of nucleosomes. The long tail of N-terminus in histones which makes the interaction between neighbor nucleosomes may be affected and undergo a variety of modifications such as lysine and arginine methylation, lysine acetylation, and serine phosphorylation (Figure 2). Histone changes affect the organization of the nucleosomes in higher order DNA packaging [11]. According to Turner, histone modifications are used for scheduling the activity of genes during the stages of differentiation [12].

Nonhistone proteins may affect the chromatin configuration through interacting with DNA and histones. For instance, ATP dependent complexes which rearrange the chromatin structure directly move and relocate nucleosomes along the DNA [13]. The second group of protein complexes propounded in epigenetics includes HP1, Polycomb, and Trithorax groups. These proteins attach to the DNA or especially modified histones and catalyze DNA methylation or other changes in histones; for example, Polycomb protein complex arrests histone methylation by interacting with DNMT and recruits DNA methylation [14]. Transcription factors can also affect the chromatin structure and be involved in the heredity of epigenetic changes during cell division [15].

In brief, the interaction between different epigenetic mechanisms controls the accessibility of genes by the transcription machinery. Epigenetic mechanisms are completely intertwined in controlling one another and the function of the target genes in an intensifying or attenuating manner which the activities of these genes can be aligned or nonaligned [16].

## 6. DNA Methyltransferase Enzymes

In mammals, DNA methyltransferase enzymes include 4 members in two families which are distinct from each other both structurally and functionally. The DNMT3 family provides the CpG methylation through de novo path, whilst the DNMT1 family maintains the methylation models during replication [17].

The DNMT3 family includes 2 enzymes DNMT3a and DNMT3b. A regulatory factor similar to DNMT3 enzyme is placed beside these enzymes which is referred to as DNMT3L [18]. DNMT3a and DNMT3b have similar domains. They both have a variable region in the N-terminus and a PWWP domain which probably affects the nonproprietary connection to DNA [19]. Despite possessing homologous structures, they have distinctly proprietary targets [20]. The rate of DNMT3b is very high at the early embryonic period and it is responsible for DNA methylation during implantation [21], whereas DNMT3a is active in the late phases of embryonic period and cell differentiation. DNMT3a also is responsible for DNA methylation in matured gametes [22, 23].

DNMT1 enzyme causes methylated models of CpG regions to be copied on new synthesized DNA. Therefore, these changes deemed as epigenetic modifications are inherited during cell division and are hereditary. Recently, obtained data from the DNMT1 structure indicate how DNMT1 connection to hemimethylated CpG region provides DNA accessibility to enzyme catalytic domain whilst link to nonmethylated CpGs protects the new-synthesized fiber by CXXC domain from its methylation. The DNMT1 activity is performed through interaction with Proliferating Cell Nuclear Antigen (PCNA) and Np95 during phase “S” of cell cycle [24, 25].

## 7. The Role of DNA Methylation and Histone Acetylation in the Regulation of Gene Expression

Epigenetic mechanisms and modifications play a crucial role in the regulation of gene expression during growth period and cell differentiation. Epigenetic mechanisms may result in gene silencing. This fact has been revealed in many types of human cancers. Despite much research that has been done in the field of epigenetics, the role of DNA methylation, histone acetylation, and deacetylation in controlling genes silencing is still not detected correctly.

Today, there are lines of evidence which demonstrate the importance of DNA methylation and histone acetylation in transcription. However, the mechanisms that cause changes in the histone acetylation and methylation of CpG sites are not completely identified. Similarly, it can be stated that the rise of epigenetic marks in human cancer remains unknown! Whilst it appears that a correct understanding is achieved for maintenance and reserving the DNA methylation patterns after replication by DNMT1 enzyme, the causes of methylated cytosine in CpG sites remain largely unknown.

It is believed that some DNA sequences are probably identified by DNA methyltransferase enzyme as target. However, there is some evidence proving that the initial DNA sequence causes cytosine methylation in CpG islands [26, 27]. Notwithstanding that the DNMT3a and DNMT3b enzymes are specific to the CpG island methylation, they seem not to be able to differentiate between the DNA sequences [28].

According to some research, DNA methylation is led by histone modifications [29, 30]. Today, we know with certainty that transcriptional silencing of genes is directly connected to the amount of histone acetylation and DNA methylation. Nevertheless, the propounded question is whether DNA hypermethylation or histones hypoacetylation leads to gene silencing or epigenetic modifications are consequences of gene silencing. Identifying events that cause inactivation of genes are critical and very important for understanding the mechanisms that lead to cancer; thus, the correct perception of these events ultimately can be helpful in cancer prevention and treatment.

Recent studies show a significant correlation between histones acetylation, DNA methylation, and gene silencing. Since then, interactions between these mechanisms are thought to be likely. In fact, implicit and explicit lines of evidence indicate the connections between histones acetylation and DNA methylation [26, 28, 31, 32]. However, it seems that there is still no general consensus that epigenetic modifications can lead to cancer inception.

## 8. Relationship between Gene Silencing and Disease

Any changes made in the genes expression play an important role in the incidence of the disease (or pathogenesis). In some cases, changes in gene expression can result from genetic defects in genes and nucleotide sequences. In some groups of patients, the aberrant epigenetic changes may alter the gene expression. As mentioned earlier, two major epigenetic mechanisms that alter and modify the gene expression are DNA methylation and histone changes [33]. Several changes in the pattern of DNA methylation and silencing the genes that cause various diseases are illustrated in Table 1.

Change in methylation pattern may lead to congenital defects. For example, downregulation of some genes affects the growth rate and changes in the DNMT3b catalytic domain results in immunodeficiency [33, 34].

## 9. Epigenetics in Normal Cells 

The genes functions in eukaryotic cells have been well-defined which can be affected by factors such as growth stage, cell periphery, or other circumstances surrounding the cell. Sequence of genes and their regulatory factors are conserved in initial DNA except for a few variables such as B and T lymphocytes. DNA and its associated proteins are subjected to covalent changes, and the location and pattern of these changes determine the growth phase and cell condition.

### 9.1. DNA Methylation

DNA methylation plays an important role in controlling cellular processes such as embryonic growth, transcription, inactivation of chromosome X, and determination of gene mapping [35].

In humans, methylation occurs in carbon 5 of cytosine which is positioned before guanine [26]. In normal cells, the CpG methylation is mainly done in the repetitive regions of genome including satellite regions, endogenous retroviruses, LINEs, and SINEs [36]. CpGs do not exist randomly in the genome, but they are located in GC rich regions referred to as CpG islands. These islands are usually found at the end of 5′ regulatory regions of many genes [37]. In normal cells, a great part of CpG islands (about 94%) is nonmethylated.

Altogether, the CpG island methylation is associated with gene silencing; it has also been shown that methylation of these islands is connected with the histones deacetylation in addition to other factors being involved in genes transcription [38]. The methylation can play a major role in reducing the gene expression as well as inactivation of the X chromosome in females and chromosome stability through hypermethylation of repetitive sequences [39].

### 9.2. Histone Changes

Up to now, over 60 histone modifications including acetylation, methylation, phosphorylation, and ubiquitylation have been identified, either by specific antibodies or spectrophotometric techniques [11]. Among all known changes, the lysine methylation shows the highest degree of compression where each methylated region might independently affect the gene activity. In general, the main function of these changes is still unknown. Transcription initiation and elongation are associated with changes of the N-terminal region of nucleosomal H3 and H4 histone proteins in the promoter region [40, 41].

Lysine acetylation is generally associated with increased accessibility of chromatin and consequently enhanced activity of transcription enzymes. But the effect of the lysine methylation on histones varies depending on the location where the histone modification has been detected [42]. In addition to regulatory role of histone changes in transcription, it also participates in replication, repair, and organization of DNA [11].

## 10. Epigenetics in Cancers

Today, there are various techniques that reveal the correlation between cancers and epigenetics. They illustrate the connection between the inactivation of microRNAs, genes, and epigenetic changes in cancer progression.

### 10.1. DNA Methylation

Change of DNA methylation pattern in CpG islands was the first and most significant abnormal epigenetic change identified in cancerous cells. There is a lot of evidence representing the DNA methylation as a change towards the beginning of the carcinogenesis processes. In particular, In particular, in age dependent changes, the DNA methylation pattern may be altered [43]. Age-related modifications can be closely related to the changes in the methylation pattern and the occurrence of cancer in a specific region of the body. Furthermore, in case of the relationship between the environment and the underlying DNA methylation, there is a strong correlation between environmental factors and development of cancer [44]. The DNA methylation pattern in cancers is various. Hypomethylation is an extremely common change in association with many cancers such as stomach, kidney, colon, pancreas, liver, and lung [45–47].

Low level methylation in cancers is substantially due to the loss of methylation at repetitive sequences as well as the introns demethylation [48]. During the development of cancer, hypomethylation degree may increase in the DNA and the progressive lesion could participate in the reproduction and metastasis of cancer cells [48].

The following 3 mechanisms have been suggested for DNA hypomethylation: (a) the increased instability of the genome, (b) the reactivation of factors that are capable of movement on DNA, and (c) the functional defect in elements related to genome [49].

Transcription of the regions with a modified methylation pattern which have resulted in hypomethylation would cause the impairment of genome. Hypomethylation of HPV genome in cervical cancer leads to progression of cancer [47]. The reverse can also occur. In such a mode, hypermethylation of tumor suppressor genes in CpG islands as well as in the micro RNA genes leads to inactivation of tumor suppressor genes. The hypermethylation of CpG islands in promoter region is a crucial incident in the initiation of carcinogenesis process.

Aberrant methylation of CpG islands is also an important factor in the proliferation of tumor cells which leads to silencing of tumor suppressor genes and molecules involved in the cell differentiation. For instance, increased methylation (hypermethylation) of CpG islands within the promoter of tumor suppressor genes is associated with various cancers such as E-cadherin, MLH1, and CDKN2A [49]. Hypermethylation of CpG islands is associated with silencing of the miRNA. miRNAs are small RNAs with typically 18 to 22 nucleotides, regulating a lot of intracellular functions such as cells reproduction, apoptosis, and cell differentiation [50–52].

### 10.2. Histone Changes

The relationship between histone changes and cancers is specified after identifying the correlation between cancer and the DNA methylation [46]. Changes in the methylation of CpG islands could result in various histone modifications and consequently different types of cancers [53]. Independent of CpG islands methylation, the histone changes can also be directly associated with cancer progression. Activity of many histone acetylase enzymes would result in deacetylation of histone H3 in the tumor suppressor gene [54]. It seems that there are various pathways that lead to histone modification and cancer development. In some cases, the links between cancers and histone modifications are clearly marked and suggest a direct relationship [55, 56].

## 11. Impacts of Epigenetic Changes on miRNAs

**Figure 3:**
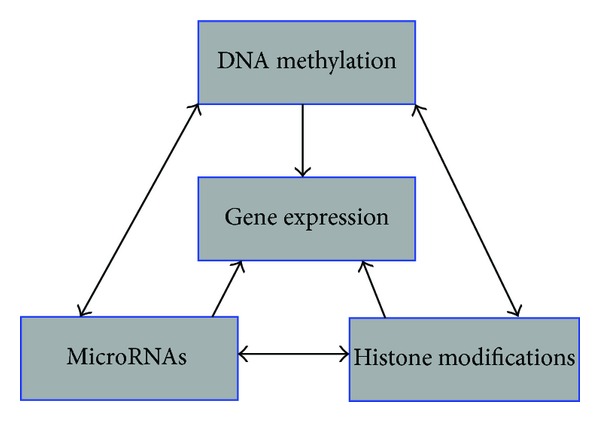
Crosstalk between genomic methylation, histone modifications, and the effects of microRNAs on gene expression.

Micro RNAs are deemed as a class of small, noncoding, and endogenous RNAs that play a crucial role in gene expression through transcription prevention by induction of a group of regulatory molecules. The miRNA can also be important in the controlling of the DNA methylation and histone modifications (Figure 3). Epigenetic mechanisms such as promoter methylation and histone acetylation may be adjusted by miRNAs expression. In some pathologic cases such as in cancers, the balance between miRNA and epigenetic processes becomes disordered. The lack of appropriate function of miRNAs and their aberrant expression are associated with the development and progression of human cancers. This process follows cell proliferation and impairment of the apoptosis [57].

Epigenetic changes on genes promoter and miRNAs dependent to the 3-UTR are considered as two important regulatory mechanisms in eukaryotics. Both DNA methylation and miRNAs can suppress the gene expression where miRNAs tend to target the genes with low DNA methylation in their promoter [58]. Recently, it has been shown that epigenetic mechanisms including DNA methylation and histone modifications affect not only gene expression but also the miRNAs. In fact, miRNAs like any other involved genes are affected by similar epigenetic regulations.

Epigenetic abnormalities can increase the rate of miRNA expression in tumor cells, and this increase may lead to the formation of these tumor cells. Almost half of miRNA genes are associated with CpG islands and their expression can be adjusted by methylation [59]. There are scientific lines of evidence suggesting that epigenetic mechanisms are responsible for regulating more than 100 miRNAs and more than half of them have been identified in the form of methylated specific cancers and about 20 kinds of tumors [60]. miRNAs can be impaired due to abnormal expression of other epigenetic regulatory mechanisms, including the HDACs (histone acetylase) or Polycomb suppressor groups [61].

Incorporation of three methyl groups on lysine 27 of histone H3 (H3k27me3) and two methyl groups on lysine 79 in histone H3 (H3k79me2) has been identified in 47 miRNAs (out of 174 miRNAs) in the colorectal cancer cells which is the result of epigenetic mechanisms [62].

Tumor cells often not only show the change in the DNA methylation pattern but also face a lot of histone changes. HDACs are overexpressed in some specific cancers such as CLL and can intervene in the silencing of several miRNA genes, including miR-15a, miR-16, and miR-29b.

## 12. Epigenetic Biomarkers in Cancer

It has been shown that DNA proprietary methylation especially in CpG islands can be used as a marker for early cancer detection, disease progression, and prediction of the response to cancer treatment [63].

There are reasons to suggest the methylated tumor suppressor genes as a good diagnostic marker. Some of these reasons include the following: (1) DNA is a stable molecule that can be easily extracted from body fluids and tissues and includes the methylation data; (2) detection of methylation change signals can be considered as an important marker to diagnose gene expression change; however identifying them in normal cells is very difficult; (3) the operating protocols for analyzing the protein or the cDNA expression level are not difficult. Epigenetic biomarkers can be easily extracted from body liquids such as blood, saliva, or urine and used to identify and diagnose tumors at the initial phase of disease [64].

Detection of methylated DNA which is derived from tumors in serum and plasma has shown similar methylation pattern in all kinds of tumors; therefore by tracing these markers, the beginning phase of tumor genesis processes could be detected [63]. Promoter methylation of gene P16 is a good marker for early detection of lung cancer. This marker is detectable and identifiable 3 years prior to the clinical symptoms of cancer [65]. The value of disease prognosis by means of DNA methylation is different in variety of cancers. For instance, in myelodysplastic syndrome, while the prognosis is very weak in the early stage of the disease, promoter methylation of genes HIC1, CDH1, ER, and P15 is diagnosable and traceable [66].

The MGMT gene silencing which is due to methylation in promoter region is an appropriate diagnostic marker in glioblastoma patients [67]. If the functions of DNMT and HDAC suppressors lead to the activation of tumor suppressor genes, then the DNA methylation would be a favorable marker for predicting response to treatment by epidrugs. Further identification of these markers is required for production of epidrugs in the future, but the overall response to the treatment in patients who are being treated by DNMT and HDACs suppressors is not significantly considerable at the present. It should be noted that the focus on the DNA methylation as a marker is essentially applied for early detection of disease and only a very few studies have been done on the assessment of the DNA methylation as a marker for examination of response to treatments by DNMT and HDACs suppressors [68]. It is also known that biomarkers such as different HDAC isoforms and histone acetylation rate are very valuable for prediction of patient response to the treatment by the HDAC suppressors [69].

## 13. Epigenetic Treatments

Upon revealing the aberrant epigenetic changes in malignant cells that lead to tumor suppressor genes silencing, the research has been expanded for achieving new drugs that are able to reactivate the silenced genes due to these changes [70].

Both genetic and epigenetic changes are factors that contribute to cancer development and progression; while the genetic changes are not reversible, epigenetic changes can return to their normal state. Reversibility of epigenetic changes caused the epigenetic treatments to be prioritized among the suggested treatments. The DNA methyltransferase and histone deacetylase enzymes have been assumed to be the most important targets of these treatments. Today, some DNMT and HDACs suppressors have been approved by Food and Drug Administration (FDA) as an anticancer treatment. The use of epigenetic targets is one of the promising ways for the treatment of cancer [71]. Azacitidine and decitabine were the first synthesized drugs for this purpose that suppress the DNMT enzyme. In 2004, FDA approved decitabine (5-aza-2-deoxycytidine) as an effective medication for myelodysplastic syndrome. The use of these drugs has remained problematic due to their volatility in water, disruption in the growth and differentiation of myeloid blood cells and side effects such as poisoning is limited [70].

Epigenetic changes allow cancer cells to adapt to their surrounding environment. However, the tumor suppressor genes that are hypermethylated can be reactivated by drugs. Reexpression of hypermethylated genes in the cancer cells is conducted by demethylating agents [72]. DNA demethylating drugs in low doses have antitumor activities. For example, drug 5-azacitidine (Vidaza) has received approval from the FDA for the treatment of leukemia [73–75]. The HDAC enzyme suppressing drugs arrest the cell cycle in differentiation stage. Although, they have demonstrated apoptotic activity *in vitro*, the specific mechanism has not yet been discovered [76].

All together, the HDAC enzyme suppressors are insignificant in the tumors treatment. Sometimes, DNA demethylating drugs and HDAC suppressors can result in unintended and contradictory consequences because of the increased gene expression. However, achieving epigenetic specific treatments, which directly target transcription factors and gene promoters, has provided a lot of hope for cancer treatment.

## 14. Conclusion

Epigenetics is a hereditable process that affects the gene expression without changing the DNA sequence. Epigenetic processes include DNA methylation and histone and chromatin changes. The importance of epigenetic modifications in cancer is well known. Hence, plenty of research has been conducted in recent years on mechanisms involved in epigenetic changes in cell. Recent advancements in field provide a precise pattern of methylation, acetylation, and miRNA level. These findings have led to the identification of biomarkers in various diseases bringing optimism and hope for curing these diseases in the near future.

## Figures and Tables

**Table 1:** Gene silencing proteins and diseases [78].

Protein	Cellular defect/disease
DNMT1	Developmental abnormalities *Igf2* imprinting Colon cancer Lymphoma Pancreatic cancer

DNMT3B	Developmental abnormalities ICF Bladder cancer Breast cancer Colon cancer Hepatocellular carcinoma Lung cancer

MeCP2	Chromosome instability/cell cycle defects Breast cancer Rett syndrome

EZH2	Cell cycle defects Barrett's esophagus Bladder cancer Breast cancer Colorectal cancer Melanoma Myeloma/lymphoma Hepatocellular carcinoma Prostate cancer Wilms tumor

Suv39h1	Blood cell defects (RBC and WBC) Chromosome instability Chromosome instability/cell cycle defects

HP1	Breast cancer Medulloblastoma Papillary thyroid carcinoma Viral latency
